# The Influence of Rootstock and High-Density Planting on Apple cv. Auksis Fruit Quality

**DOI:** 10.3390/plants10061253

**Published:** 2021-06-20

**Authors:** Kristina Laužikė, Nobertas Uselis, Giedrė Samuolienė

**Affiliations:** Institute of Horticulture, Lithuanian Research Centre for Agriculture and Forestry, Kauno 30, Babtai, LT-54333 Kaunas distr., Lithuania; nobertas.uselis@lammc.lt (N.U.); giedre.samuoliene@lammc.lt (G.S.)

**Keywords:** *Malus domestic*, carbohydrates, organic acids, mineral elements

## Abstract

Global demand for food is increasing each year, but the area of land suitable for farming is limited. Thus, there is a need to grow not only larger quantities of food but also higher quality food products in the same area. This study aimed to evaluate the influence of rootstock and high-density orchards on cv. Auksis fruit quality. Two rootstocks were selected for this experiment, P 22 super dwarfing and P 60 dwarfing. Apple trees cv. Auksis were planted in the year 2001 in single rows spaced 1.00 m, 0.75 m, and 0.50 m, apart with 3 m between rows. High-density planting and rootstock combination was found to have no significant effect on sugar accumulation and most of the elements in apple fruits. However, super dwarfing P 22 rootstock accumulated significantly higher (up to 45%) content of organic acids and up to 33%–44% lower DPPH free radical scavering activity compared to P 60 dwarfing rootstock. After summarizing the obtained results, apples which accumulated the most antioxidants (according to the activity of phenolic compounds, DPPH^•^ and ABTS^•^), magnesium, and potassium were collected from cv. Auksis apple trees which was grafted on super dwarfing P 22 rootstock and planted at 3 × 0.75 m distances.

## 1. Introduction

Global demand for food is increasing each year, but the area of land suitable for farming is limited. Land degradation leads to smaller areas suitable for good yields [1]. Thus, there is a need to grow not only larger quantities of food but also higher quality food products in the same area. High quality products reduce the quantitative mass requirements, as more nutrients can be obtained from the same amount. We can also use trees to grow valuable and high-quality food [2]. At the same time, trees can be used to stop the processes of land degradation and preserve its productivity. Over the last five years, world apple yields have ranged from 83 to 87 million tons per year. In 2019, apples ranked second after bananas among fruits in terms of the highest yields [3].

To fill the area faster and get a higher yield per unit area, world growers have introduced high-density planting schemes in apple orchards [4,5]. Increasing the density of trees results the more efficient land use and higher yields per area. However, increasing tree canopy during growth results in competitive stress. The increased density leads to decreased yields from the tree, but yields per unit area remain higher [6,7,8,9]. Seeking to grow high-density apple orchards efficiently, it is important to select the distances between the trees and the corresponding vigor rootstocks. Different rootstocks affect canopy growth intensity differently. According to the tree vigor, apple rootstocks are categorized as super dwarfing, dwarfing, semi-dwarfing, semi-vigorous, and vigorous. Dwarfing rootstocks are mainly planted in high-density orchards due to their smaller canopy and compact tree roots [10,11,12].

A good combination of planting scheme and rootstock can result in a high yield and high-quality apple fruits suitable for the consumer [9,10]. Apples are widely used all over the world due to their good properties like good chemical composition and excellent shelf life. The excellent ratio of organic acids (0.2–0.8%) and sugars (10–12%) in the fruit is not only beneficial but also gives a pleasant taste to the consumer [9,13,14]. Also, apples are one of the most phenolic-accumulating fruits [15], which act as an antioxidant, inhibit inflammatory processes [16], and have anti-cancer [17] and anti-aging [18] effects. In addition to the apple bioactive compounds already mentioned, they are also rich in macro (Ca, Mg, K, Na) and micro (Zn, Fe, Mn, Cu) elements [9,19,20]. Although apples have excellent properties, their chemical composition varies and depends on various factors. One of the main factors upon which the characteristics of apples depend is the variety [15,21,22]. Another factor that determines both the quantity and the quality of fruits is a properly selected rootstock [10,23,24]. With the right combination of variety and rootstock, we have a tree programmed for a high-quality harvest. However, a well-chosen rootstock alone is not enough; the environmental conditions also change the composition and quality of the fruit. Sunlit canopies result in more active photosynthetic processes [8,25]. With insufficient sunlight, glucose and sorbitol produced during photosynthesis are transported to the apple fruit, where they can be stored as fructose, glucose, malic acid, or starch [13,26,27]. Increasing the density of the trees decreased the penetration of the sun through the canopy, increasing their shading of each other. So, as the density of fruit trees increases, the light penetration in the crown decreases, the photosynthetic properties of apples deteriorate, the total sugar content in the fruit decreases, but the phenolic compounds increase [7,9].

In previous studies of apple quality in high-density orchards the data are often narrow and do not describe changes in the chemical composition of the fruit. Therefore, the aim of this study was to evaluate the influence of rootstock and high-density orchards on cv. Auksis fruit quality.

## 2. Results

**Figure 1:**
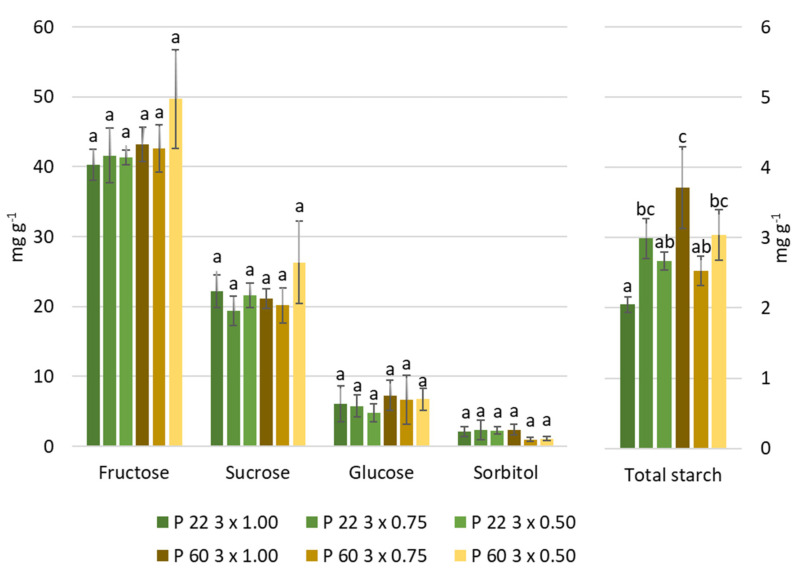
The effect of distances and rootstock on carbohydrates content in apple fruits. The mean value (n = 3 × 3 = 9 mg g^−1^ fresh weight) ± standard deviation is presented. The data were processed using a two-way analysis of variance (ANOVA), the Tukey (HSD) test at the confidence level *p* = 0.05. The different letters in blocks indicate significant differences.

Basic fruit sugars were not significantly affected either by the rootstock or by the planting scheme, however, the sorbitol content in the fruit increased in P 22 super-dwarfing rootstock up to 45% compared to the P 60 dwarfing rootstock (Table S1). Higher starch content was found in the fruit of apples grafted on P 60 rootstock, at 1 m between trees, meanwhile, lower content was found in the fruit of apples grafted on P 22 rootstock at the same distances (Figure 1). More vigorous P 60 rootstock reduced sorbitol accumulation by 34% and increased accumulation of starch by 20% compared with super-dwarfing P 22 rootstock.

Significant differences in the amount of total phenols in apples were found in the interaction between rootstock and distance (Table 1). Significantly, the lowest content of phenols was found in the P 22 dwarfing rootstock apples planted at distances of 1 m, while the highest amounts of total phenols were accumulated in fruits with P 22 rootstock planting distance 0.75 m and P 60 rootstock planting distance 1 m. The distances, rootstock, or their interaction had no significant effect on ABTS free radical scavenging activity in apple fruits. While DPPH was significantly affected by the rootstock, with P 22 super-dwarfing rootstock DPPH free radical scavenging activity was 33%–44% lower than with P 60 dwarfing rootstock.

**Figure 2:**
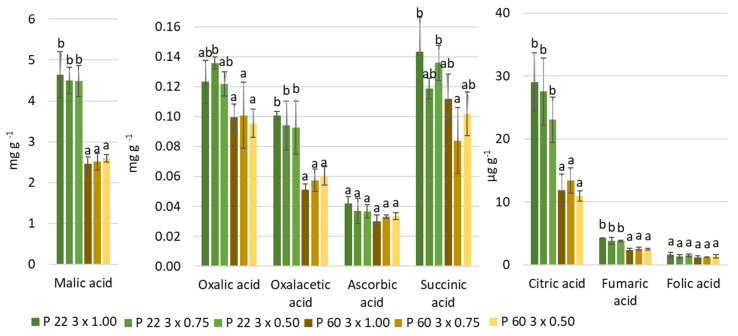
The effect of distances and rootstock on organic acids in apple fruits. The mean value (n = 3 × 3 = 9) ± standard deviation is presented. The data were processed using two-way analysis of variance (ANOVA), the Tukey (HSD) test at the confidence level *p* = 0.05. The different letters in blocks indicate significant differences.

The main organic acid was determined in cv. Auksis in apples was malic acid, the amount varied between 2.5–4.6 mg g^−1^. Malic acid was followed by oxalic (0.10—0.14 mg g^−1^), oxalacetic (0.05–0.10 mg g^−1^), ascorbic (0.03–0.04 mg g^−1^), succinic (83.6–143.4 µg g^−1^), citric (11.0–29.0 µg g^−1^), fumaric (2.5–4.3 µg g^−1^) and folic (1.2–1.7 µg g^−1^) (Table S2). The rootstock had the greatest significant effect on the content of organic acids in the fruits. Less vigorous super dwarfing P 22 rootstock accumulated significantly higher (up to 45%) content of almost all identified organic acids compared to dwarfing P 60 rootstock (Figure 2).

The main element found in apples was potassium (0.623–0.730 mg g^−1^), followed by calcium (0.094–0.155 mg g^−1^), magnesium (0.063–0.071 mg g^−1^) and sodium (0.017–0.022 mg g^−1^) (Table 2). Significantly the highest amounts of potassium and magnesium were found in fruits, where apple trees were grafted on P 22 rootstock and grown at 3 × 0.75 m distances. Meanwhile, higher amounts of sodium and magnesium were found in fruits where apple trees were grafted on P 60 rootstock, regardless of the distance between trees.

The highest content of minor elements was found in the following order: iron (0.99–2.60 µg g^−1^); followed by manganese (about 1 µg g^−1^); copper (0.609–0.890 µg g^−1^); and zinc (0.275–0.358 µg g^−1^) (Table 3). The manganese content in fruits was not significantly affected by rootstock, planting distance, or their interaction. Meanwhile, the lowest amounts of iron and copper were accumulated in the fruits, where trees were planted at the largest distances and drafted on P 22 rootstock. Fruits from apple trees grafted on P 60 dwarfing rootstock accumulated 2.4–2.6 times bigger amounts of iron and up to 46% more copper compared to fruits from apple trees grafted on P 22 super dwarfing rootstock.

## 3. Discussion

The entry of the sun into the canopy has a strong effect on photosynthesis processes, the higher density of trees reduces the penetration of light into the lower and inner layers of the canopy, which results in a decrease in photosynthesis rates [28,28]. Previous data showed that increased cv. Auksis tree density negatively affected photosynthesis intensity and other indices related with photosynthetic response [7,8]. The carbohydrates produced in the leaves are further transported to the fruit, so the amount of carbohydrates in the fruit strongly depends on the production in the leaves [13,26,27]. Our study confirmed that the planting density did not have a significant effect on the accumulation of sugars in the leaves of the apple tree cv. Auksis [8], and neither on the amount of sugars in the fruit (Table 1). P 60 dwarfing rootstock and P 22 super dwarfing rootstock had different effects on carbohydrate content in fruits; significant differences were found for sorbitol and starch content (Table 1). MM 106 rootstock increased carbohydrate accumulation compared to M 9 rootstock [29], and super dwarfing P 22 rootstock increased sorbitol, glucose, and fructose accumulation compared to dwarfing rootstocks M 9, M 26, P 67, and B 396 [30]. However, both P 22 and P 60 are low-vigorous rootstocks, which may lead to differences in carbohydrate accumulation that are not so pronounced as between more vigorous rootstocks, such as M 26 or B 396 [29,30].

Competitive stress for light caused by the high-density canopy from mechanical pruning has been shown to result in an increase of total phenol content in the fruit [9,31]. On the other hand, according to Kviklys et al. [32], rootstock also had the significant effect on phenol accumulation. However, no significant differences between the rootstock or the planting distances were found, but the interaction between rootstock and planting distance highlighted significant differences in phenol accumulation. More vigorous rootstock P60 planted at 1 m distance between trees resulted in a significant increase in fruit total phenols, while the highest amounts of total phenols in less vigorous P 22 rootstock was obtained with 0.75 m planting distances. (Table 1). According Sethi et al., antioxidant activity strongly correlated with the amount of phenolic compounds [33]. The content of phenolics, and therefore antioxidant activity, can be influenced by various factors such as pre-harvest meteorological conditions, cultural practices, maturity, and harvesting methods [34,35]. DPPH scavenging activity decreased from 44–20 mM Trolox (equivalent) g^−1^ (depending on variety) to 11.7–3.6 mM Trolox g^−1^ (depending on variety) during fruit ripening, while ABTS scavenging activity from 4.5–2.6 mM Trolox g^−1^ to 1.4–0.9 mM Trolox g^−1^ [35]. Significantly lower starch content (Figure 1) and DPPH antioxidant radical scavenging activity (Table 1) in fruits from apple trees grafted on P 22 super dwarfing rootstock was found. Thus, it can be presumed that fruits harvested at the same time on P 22 super-dwarfing rootstock had a higher ripening level than fruits from trees grafted on P 60 dwarfing rootstock.

The main organic acid in apples is malic acid, its content can vary in range from 2.73 mg L^−1^ to 7.07 mg L^−1^ of juice depending on the variety [36]. The content of malic acid in cv. Auksis fruits varied from 2.46 mg g^−1^ to 4.64 mg g^−1^ depending on planting density and rootstock (Figure 2). According to Tomala et al. [37], significant influence of rootstock on the acidity of apples does not occur every year. Evaluating the three-year data, we found that the rootstock had a significant effect on almost all analyzed organic acid accumulation (Figure 2). P 22 super dwarfing rootstock resulted a significant increase in all tested organic acids compared to P 60 dwarfing rootstock.

Dietary recommendations suggest to consume 4.7 g of potassium per day, moreover, the prevention from cardiovascular diseases may be associated with higher consumption [38]. The content of potassium in cv. Auksis fruit varied from 0.6 to 0.7 mg in FW (Table 2), which means that in an average apple (~ 150 g) between 90 and 105 mg of potassium is accumulated. Auksis fruits were high in calcium as well, it is one of the most important elements in the human body. The amounts of these elements in the fruit is determined by various factors, such as transpiration, uptake of water and nutrients from the soil, and chemicals sprayed on the fruit trees [39,40]. The amount of elements accumulated in apples strongly depends on its root and its ability to absorb substances from the soil [20]. Super-dwarf rootstock P 22 forms a smaller root than the dwarf rootstock P 60, so its ability to absorb substances from the soil is lower. Our data (Table 2 and Table 3) are in agreement with Kucukyumuk and Erdal [41] and Reig et al. [42], which showed that amounts of both major and minor elements were significantly influenced by rootstock. Assessing the accumulation of micro and macro elements in the fruit, it can be concluded that for low-growing rootstocks P 60 and P 22 grown at the distances studied in our experiment, there was a complete supply of nutrient area, as no significant differences were found between the planting distances.

## 4. Materials and Methods

### 4.1. Plant Material and Growing Conditions

A trial was carried out in an experimental intensive orchard in Lithuania, (55°60ʹ N, 23°48ʹ E) in 2017–2019. Apple samples were collected from fully grown, canopy-formed, and root-covered trees that strongly feel competitive tension, not only due to light but also water and nutrients. The apple tree (Malus domestica Borkh.) cv. Auksis was grafted on P 60 dwarfing rootstock and P 22 super dwarfing rootstock. Apple trees were planted in single rows spaced 1.00 m, 0.75 m, and 0.50 m apart, with 3 m between rows. Pest and disease management was carried out according to integrated plant protection practices, and the orchard was not irrigated. The soil conditions of the experimental orchard were clay loam, pH 7.3, humus 2.8%, P_2_O_5_ 255 mg kg^−1^, and K_2_O 230 mg kg^−1^. The experiment was arranged in three replicates of five trees, with replicates arranged in complete randomization. Three trees from the replicate were selected for sampling. The samples were collected from the whole canopy using full randomization. Five apples were taken from three trees in one replicate at harvest time on commercial ripening (BBCH 87−88). Each apple was divided into four parts, the seed hopper was removed, and a sample was taken from each part of the pulp. The composite sample of five apples was weighed for further analysis. The remaining apples were crushed and dried to air-dry weight to determine the elemental composition.

### 4.2. Determination of Soluble Sugars by Ultra-Fast Liquid Chromatography (UFLC)

Soluble sugar (fructose, glucose, sucrose, and sorbitol) contents were evaluated using the UFLC method with evaporative scattering detection (ELSD) [43]. About 1 g of fresh plant tissue was ground and diluted with deionized water. The extraction was carried out for 4 h at room temperature, centrifuged at 14,000× *g* rpm for 15 min. A cleanup step was performed before the chromatographic analysis: 1 mL of the supernatant was mixed with 1 mL 0.01% (*w*:*v*) ammonium acetate in acetonitrile and incubated for 30 min at 4° C. After incubation, samples were centrifuged at 14,000× *g* rpm for 15 min and filtered through 0.22 µm PTFE syringe filter (VWR International, Radnor, PA, USA). Analysis was performed on a Shimadzu Nexera UPLC (Kyoto, Japan) system. Separation was performed on a Supelcosil 250 × 4 mm NH_2_ column (Supelco, Bellefonte, PA, USA) using 77% acetonitrile as the mobile phase at 1 mL min^−1^ flow rate. The calibration method (R^2^ < 0.99) was used for each individual sugar quantification (mg g^−1^ in FW).

### 4.3. Determination of Total Starch by Spectrophotometric Method

The total starch content was determined using the total starch Megazyme assay kit (Megazyme International Ireland Limited, Wicklow, Ireland), a total starch assay kit based on the use of thermostable a-amylase and amyloglucosidase method of determination of starch in samples which also contains D-glucose and/or maltodextrins.

### 4.4. Determination of Total Phenolic Compounds by Spectrophotometric Method

Using a colorimetric method, the total content of phenolic compounds was determined using methanol extracts—1 g of plant tissues grounded with liquid nitrogen and diluted with 10 mL of 80% methanol. The extract was mixed and left for 24 h in the fridge ( + 4 °C), subsequently centrifuged at 5000× *g* rpm for 5 min. Next, 0.1 mL of extract was diluted with 0.2 mL 10% Folin-Ciocalteau reagent (Folin reagent diluted with bi-distilled water 1:10) and with 0.8 mL 7.5% Na_2_CO_3_ solution [44]. The absorbance was measured after 20 min at 765 nm using a Genesys 6 spectrophotometer (Thermospectronic, Waltham, MA, USA) against distilled water as a blank. The results were expressed as mg of gallic acid equivalent per g fresh weight (FW).

### 4.5. Determination of DPPH Free Radical Scavenging Activity by Spectrophotometric Method

The antioxidant activity of methanol extracts of the investigated plants was evaluated spectrophotometrically relating to the 2,2-diphenyl-1-picrylhydrazyl (DPPH^•^) free radical scavenging capacity. 1 g of plant tissues grounded with liquid nitrogen and diluted with 10 mL of 80% methanol. The extract was mixed and left for 24 h in the fridge (4 °C) and subsequently centrifuged at 5000× *g* rpm for 5 min. 1 mL DPPH^•^ solution (60µ DPPH^•^) and 0.1 mL for extract add into a cuvette. The absorbance scanned after 16 min from the beginning of the reaction at 515 nm [45].

### 4.6. Determination of the ABTS Radical Scavenging Activity by Spectrophotometric Method

The ABTS^•^ (2.2′-azino-bis (3-ethylbenzothiazoline-6-sulfonic acid) diammonium salt) scavenging activities of apple extracts were determined. The same extraction was used as for the determination of DPPH free radical scavenging capacity. ABTS^•^ was dissolved in methanol at a concentration of 2 mM. The ABTS radical cation was produced by incubating the ABTS^•^ stock solution with 200 μL K_2_S_2_O_8_ (0.1982 g/10 mL H_2_O) in the dark for 16 h. Following this, 100 μL of the diluted sample was mixed with 2 mL of ABTS^•^ solution and the absorbance was scanned for 11 min (plateau phase) at 734 nm. The ABTS^•^ scavenging activity of each extract was calculated as the difference between the initial absorbance and that after reacting for 10 min, which was expressed as mmol (ABTS^•^) scavenged per 1 g fresh sample (mmol g^−1^). Methanol was used as the blank solution [46].

### 4.7. Determination of Organic Acid by High-Performance Liquid Chromatography (HPLC)

Organic acid (oxalic, malic, citric, and succinic) contents were determined using the HPLC method [47] on Shimadzu 10A (Japan) system with diode-array detector (DAD). The sample was prepared grinding plant material and diluting with H_2_O 1:10 (*w*:*v*). Extraction was performed in a heated water bath (50 °C) for 30 min. The extract was clarified by centrifugation at 10,000 rpm for 15 min and filtered through a 0.22 µm PTFE syringe filter (VWR International, Radnor, PA, USA). Separation was performed on Lichrosorb RP-184.6 × 250 mm, 5µm column (Altech). Mobile phase—0.05 M sulphuric acid, flow rate 0.5 mL min^−1^, injection volume—10 µL. The calibration method (R^2^ < 0.99) was used for each organic acid quantification (mg g^−1^ in FW).

### 4.8. Determination of micro- and macro- elements by Inductively Coupled Plasma-Optical Emission Spectrometry (ICP-OES)

The macro and microelements contents in microgreens were determined by microwave digestion technique combined with inductively coupled plasma optical emission spectrometry [48,49]. Complete digestion of dry microgreen material (0.5 g) was achieved with 100% HNO_3_ using microwave digestion system Multiwave GO (Anton Paar GmbH, Graz, Austria). The digestion program was as follows: 170 °C was reached within 5 min, digested for 20 min; the mineralized samples were diluted to 50 mL with deionized water; and the elemental profile was analyzed using an ICP-OES spectrometer (Spectro Genesis, SPECTRO Analytical Instruments, Kleve, Germany). The operating conditions employed for ICP-OES determination were 1300W RF power, 12 L min^−1^ plasma flow, 1.0 L min^−1^ auxiliary flow, 0.8 L min^−1^ nebulizer flow, 1.0 mL min^−1^ sample uptake rate. The analytical wavelengths (nm) chosen were: B ^+^ 249.773 nm, Ca^2+^ 445.478 nm, Cu^+^ 324.754 nm, Fe^2+^ 259.941 nm, K^+^ 766.491 nm, Mg^2+^ 279.079 nm, Mn^2+^ 259.373 nm, Na^+^ 589.592 nm, P^+^ 213.618 nm, S^+^ 182.034 nm, and Zn^+^ 213.856 nm. The calibration standards were prepared by diluting a stock multi-elemental standard solution (1000 mg L^−1^) in 6.5% (*v*/*v*) nitric acid, and by diluting stock phosphorus and sulfur standard solutions (1000 mg L^−1^) in deionized water. The calibration curves for all the studied elements were in the range of 0.01–400 mg L^−1^.

### 4.9. Statistical Analysis

MS Excel Version 2010 and XLStat 2020 Data Analysis and Statistical Solution for Microsoft Excel (Addinsoft, Paris, France) statistical software were used for data processing. Analysis of variance (ANOVA) was carried out along with Tukey multiple comparisons test for statistical analyses, *p* ≤ 0.05.

### 4.10. Meteorological Conditions

The meteorological data were collected from “iMetos” meteorological station at the Institute of Horticulture, Lithuania research center for agriculture and forestry, Lithuania in 2017–2019. Meteorological conditions fluctuated around the average multiannual conditions during the study year (Figure S1). The precipitation increase, observed in August 2019, was obtained due to brief rainfall.

## 5. Conclusions

The highest accumulation of phenolic compounds were found in fruits of cv. Auksis trees grafted on P 22 rootstock at planting distance 0.75 m and grafted on more vigorous P 60 rootstock at planting distance 1 m between trees. Also, less vigorous P 22 super dwarfing rootstock accumulated significantly higher (up to 45%) content of almost all identified organic acids compared to P 60 dwarfing rootstock. Planting density and rootstock combinations had no significant effect on the amount of sugars identified in fruits of cv. Auksis. Significantly the highest amounts of potassium and magnesium, also, were found in fruits of trees grafted on P 22 rootstock, planted at 3 × 0.75 m distances. However, fruits of apple trees grafted on P 60 dwarfing rootstock accumulated 2.5 times more iron and up to 46% more copper compared to P 22 super dwarfing rootstock. Generally, if seeking to obtain a high-quality fruit harvest in high-density apple cv. Auksis orchards, P 22 super dwarf rootstock at 3 × 0.75 m planting distances is recommended.

## Figures and Tables

**Table 1:** The effect of distances and rootstock on total phenols and DPPH and ABTS radical scavenging activity in apple fruits. The mean value (n = 3 × 3 = 9) ± standard deviation is presented. The data were processed using a two-way analysis of variance (ANOVA), the Tukey (HSD) test at the confidence level *p* = 0.05. The different letters in blocks indicate significant differences.

Rootstock	Planting Density	Total Phenols	ABTS	DPPH
		mg g^−1^	mM TE g^−1^	mM TE g^−1^
**P22**	3 × 1.00	2.47 ± 0.40 ^a^	6.15 ± 0.95 ^a^	2.15 ± 0.15 ^a^
**P22**	3 × 0.75	3.11 ± 0.15 ^b^	8.43 ± 0.97 ^a^	2.87 ± 0.69 ^a^
**P22**	3 × 0.5	2.66 ± 0.05 ^ab^	6.72 ± 0.19 ^a^	2.32 ± 0.32 ^a^
**P60**	3 × 1.00	2.99 ± 0.13 ^b^	7.37 ± 1.08 ^a^	3.78 ± 0.36 ^b^
**P60**	3 × 0.75	2.71 ± 0.05 ^ab^	6.86 ± 0.64 ^a^	3.19 ± 0.22 ^b^
**P60**	3 × 0.5	2.74 ± 0.06 ^ab^	8.17 ± 1.91 ^a^	3.45 ± 0.26 ^b^
Effect of planting density				
	3 × 1.00	2.73 ± 0.39 ^a^	6.76 ± 1.13 ^a^	2.96 ± 1.46 ^a^
	3 × 0.75	2.91 ± 0.24 ^a^	7.64 ± 1.13 ^a^	3.03 ± 0.86 ^a^
	3 × 0.5	2.70 ± 0.07 ^a^	7.44 ± 1.45 ^a^	2.88 ± 1.20 ^a^
Effect of rootstock				
**P22**		2.74 ± 0.36 ^a^	7.10 ± 1.23 ^a^	2.45 ± 0.51 ^a^
**P60**		2.81 ± 0.15 ^a^	7.47 ± 1.28 ^a^	3.47 ± 0.35 ^b^

**Table 2:** The effect of planting distances and rootstock on major elements in cv. Auksis apple fruits. The mean value (n = 3 × 3 = 9) ± standard deviation is presented. The data were processed using a two-way analysis of variance (ANOVA), the Tukey (HSD) test at the confidence level *p* = 0.05. The different letters in blocks indicate significant differences.

		K	Ca	Mg	Na
		mg g^−1^	mg g^−1^	mg g^−1^	mg g^−1^
**P22**	3 × 1.00	0.669 ± 0.03 ^ab^	0.094 ± 0.001 ^a^	0.064 ± 0.001 ^a^	0.017 ± 0.001 ^a^
**P22**	3 × 0.75	0.730 ± 0.03 ^b^	0.102 ± 0.002 ^a^	0.071 ± 0.002 ^b^	0.019 ± 0.002 ^ab^
**P22**	3 × 0.50	0.666 ± 0.02 ^ab^	0.097 ± 0.001 ^a^	0.063 ± 0.001 ^a^	0.017 ± 0.003 ^a^
**P60**	3 × 1.00	0.659 ± 0.06 ^ab^	0.153 ± 0.002 ^b^	0.068 ± 0.002 ^ab^	0.021 ± 0.008 ^bc^
**P60**	3 × 0.75	0.624 ± 0.03 ^a^	0.155 ± 0.002 ^b^	0.063 ± 0.002 ^a^	0.020 ± 0.009 ^bc^
**P60**	3 × 0.50	0.675 ± 0.02 ^ab^	0.153 ± 0.002 ^b^	0.064 ± 0.003 ^a^	0.022 ± 0.003 ^c^
Effect of planting density					
	3 × 1.00	0.664 ± 0.042 ^a^	0.124 ± 0.035 ^a^	0.066 ±0.003 ^a^	0.020 ± 0.002 ^a^
	3 × 0.75	0.677 ± 0.063 ^a^	0.129 ± 0.031 ^a^	0.067 ± 0.004 ^a^	0.020 ± 0.002 ^a^
	3 × 0.50	0.671 ± 0.018 ^a^	0.126 ± 0.031 ^a^	0.064 ± 0.002 ^a^	0.020 ± 0.003 ^a^
Effect of rootstock					
**P22**		0.688 ± 0.039 ^b^	0.097 ± 0.004 ^a^	0.066 ± 0.038 ^a^	0.018 ± 0.014 ^a^
**P60**		0.652 ± 0.041 ^a^	0.154 ± 0.014 ^b^	0.065 ± 0.031 ^a^	0.022 ± 0.010 ^b^

**Table 3:** The effect of planting distances and rootstock on minor elements in apple fruits. The mean value (n = 3 * 3 = 9) ± standard deviation is presented. The data were processed using a two-way analysis of variance (ANOVA), the Tukey (HSD) test at the confidence level *p* = 0.05. The different letters in blocks indicate significant differences.

		Fe	Mn	Cu	Zn
		µg g^−1^	µg g^−1^	µg g^−1^	µg g^−1^
**P22**	3 × 1.00	0.99 ± 0.04 ^a^	1.04 ± 0.02 ^a^	0.609 ± 0.039 ^a^	0.283 ± 0.015 ^a^
**P22**	3 × 0.75	1.11 ± 0.02 ^a^	1.04 ± 0.07 ^a^	0.757 ± 0.047 ^b^	0.292 ± 0.009 ^ab^
**P22**	3 × 0.5	1.05 ± 0.07 ^a^	1.02 ± 0.05 ^a^	0.731 ± 0.013 ^b^	0.275 ± 0.027 ^a^
**P60**	3 × 1.00	2.39 ± 0.50 ^b^	1.04 ± 0.05 ^a^	0.881 ± 0.012 ^c^	0.317 ± 0.027 ^abc^
**P60**	3 × 0.75	2.60 ± 0.04 ^b^	1.02 ± 0.06 ^a^	0.846 ± 0.027 ^c^	0.358 ± 0.034 ^c^
**P60**	3 × 0.5	2.57 ± 0.12 ^b^	1.07 ± 0.02 ^a^	0.890 ± 0.017 ^c^	0.347 ± 0.013 ^bc^
	Effect of planting density				
	3 × 1.00	1.69 ± 0.832 ^a^	1.04 ± 0.03 ^a^	0.745 ± 0.152 ^a^	0.300 ± 0.027 ^a^
	3 × 0.75	1.85 ± 0.815 ^a^	1.03 ± 0.06 ^a^	0.802 ± 0.059 ^b^	0.325 ± 0.043 ^a^
	3 × 0.5	1.81 ± 0.837 ^a^	1.04 ± 0.04 ^a^	0.810 ± 0.088 ^b^	0.311 ± 0.044 ^a^
	Effect of rootstock				
	P22	1.05 ± 0.07 ^a^	1.04 ± 0.05 ^a^	0.699 ± 0.075 ^a^	0.283 ± 0.018 ^a^
	P60	2.52 ± 0.27 ^b^	1.04 ± 0.05 ^a^	0.872 ± 0.027 ^a^	0.341 ± 0.029 ^a^

## Supplementary Materials

https://www.mdpi.com/article/10.3390/plants10061253/s1. Figure S1 Meteorological conditions in the test year (2017—2019) and perennials (100-year average): A—temperature, B—precipitation; Table S1 The effect of distances and rootstock on carbohydrates content in apple fruits. The mean value (n = 3 × 3 = 9 mg g^−1^ fresh weight) ± standard deviation is presented. The data were processed using two-way analysis of variance (Anova), the Tukey (HSD) test at the confidence level *p* = 0.05. The different letter in blocks indicate significant differences; Table S2 The effect of distances and rootstock on organic acids in apple fruits. The mean value (n = 3 × 3 = 9)± standard deviation is presented. The data were processed using two-way analysis of variance (Anova), the Tukey (HSD) test at the confidence level *p* = 0.05. The different letter in blocks indicate significant differences.
