# Supplementary material for: The Influence of Rootstock and High-Density Planting on Apple cv. Auksis Fruit Quality
Source: Plants (Basel). 2021 Jun 20;10(6):1253. doi: 10.3390/plants10061253 (PMC8234605; doi:10.3390/plants10061253)
Supplement: Supplementary file 1 [file plants-10-01253-s001.zip › plants-1243614-supplementary.pdf]

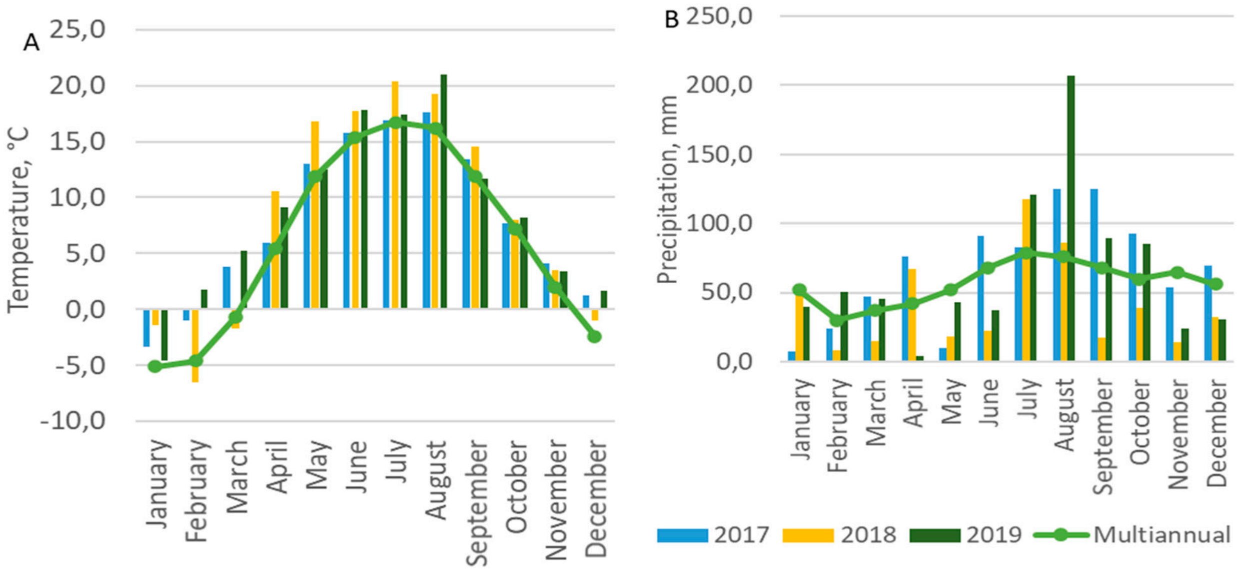

Figure S1 Meteorological conditions in the test year (2017-2019) and perennials (100-year average): A - temperature, B - precipitation

Table S1 The effect of distances and rootstock on carbohydrates content in apple fruits. The mean value (n=3*3=9 mg g^{-1} fresh weight) ± standard deviation is presented. The data were processed using two-way analysis of variance (Anova), the Tukey (HSD) test at the confidence level p = 0.05. The different letter in blocks indicate significant differences.

Rootstock	Distance	Fructose	Sucrose	Glucose	Sorbitol	Total starch
		mg g^{-1}	mg g^{-1}	mg g^{-1}	mg g^{-1}	mg g^{-1}
**P 22**	3 x 1.00	40,27±2,24^{a}	22,22±2,35^{a}	6,11±2,58^{a}	2,11±0,71^{a}	2,05±0,11^{a}
	3 x 0.75	41,62±3,90^{a}	19,38±2,10^{a}	5,79±1,63^{a}	2,38±1,38^{a}	2,99±0,28^{bc}
	3 x 0.50	41,34±1,07^{a}	21,57±1,75^{a}	4,78±1,30^{a}	2,28±0,55^{a}	2,67±0,13^{ab}
**P 60**	3 x 1.00	43,17±2,44^{a}	21,18±1,41^{a}	7,31±2,11^{a}	2,36±0,75^{a}	3,71±0,58^{c}
	3 x 0.75	42,61±3,39^{a}	20,17±2,52^{a}	6,65±3,52^{a}	0,98±0,30^{a}	2,53±0,21^{ab}
	3 x 0.50	49,70±7,06^{a}	26,31±5,89^{a}	6,74±1,60^{a}	1,12±0,24^{a}	3,04±0,36^{bc}
Effect of planting density						
	3 x 1.00	41,72±2,63^{a}	21,70±1,82^{a}	6,71±2,21^{a}	2,24±0,67^{a}	2,88±0,99^{a}
	3 x 0.75	42,12±3,31^{a}	19,78±2,12^{a}	6,22±2,49^{a}	1,68±1,18^{a}	2,76±0,33^{a}
	3 x 0.50	45,52±6,43^{a}	23,94±4,67^{a}	5,76±1,69^{a}	1,70±0,74^{a}	2,85±0,32^{a}
Effect of rootstock						
**P 22**		41,08±2,39^{a}	21,06±2,21^{a}	5,56±1,76^{a}	2,26±0,83^{a}	2,57±0,45^{a}
**P 60**		45,16±5,34^{a}	22,56±4,35^{a}	6,90±2,22^{a}	1,49±0,78^{b}	3,09±0,63^{b}

Table S2 The effect of distances and rootstock on organic acids in apple fruits. The mean value (n=3*3=9) ± standard deviation is presented. The data were processed using two-way analysis of variance (Anova), the Tukey (HSD) test at the confidence level p = 0.05. The different letter in blocks indicate significant differences.

**Rootstock**	**Distance**	**Oxalic acid**	**Oxalacetic acid**	**Malic acid**	**Ascorbic acid**	**Folic acid**	**Citric acid**	**Succinic acid**	**Fumaric acid**
		mg g^{-1}	mg g^{-1}	mg g^{-1}	mg g^{-1}	µg g^{-1}	µg g^{-1}	µg g^{-1}	µg g^{-1}
**P 22**	3 x 1.00	0,123±0,014^{ab}	0,101±0,003^{b}	4,64±0,56^{b}	0,042±0,004^{a}	1,65±0,33^{a}	29,01±4,64^{b}	143,44±22,76^{b}	4,26±0,06^{b}
	3 x 0.75	0,136±0,004^{b}	0,094±0,016^{b}	4,50±0,33^{b}	0,037±0,008^{a}	1,34±0,29^{a}	27,51±5,35^{b}	118,93±6,91^{ab}	3,81±0,55^{b}
	3 x 0.50	0,122±0,008^{ab}	0,093±0,018^{b}	4,48±0,38^{b}	0,037±0,004^{a}	1,51±0,21^{a}	23,05±3,64^{b}	135,89±11,66^{b}	3,79±0,15^{b}
**P 60**	3 x 1.00	0,100±0,009^{a}	0,051±0,004^{a}	2,46±0,17^{a}	0,030±0,004^{a}	1,22±0,24^{a}	11,82±2,59^{a}	112,00±16,30^{ab}	2,41±0,23^{a}
	3 x 0.75	0,101±0,022^{a}	0,057±0,008^{a}	2,51±0,20^{a}	0,033±0,001^{a}	1,22±0,06^{a}	13,40±2,02^{a}	83,86±22,07^{a}	2,57±0,21^{a}
	3 x 0.50	0,096±0,009^{a}	0,060±0,006^{a}	2,59±0,09^{a}	0,034±0,002^{a}	1,34±0,27^{a}	10,97±0,81^{a}	101,89±14,54^{ab}	2,47±0,14^{a}
					Effect of planting density				
	3 x 0.75	0,118±0,024^{a}	0,076±0,023^{a}	3,51±1,12^{a}	0,035±0,006^{a}	1,28±0,20^{a}	20,45±8,54^{a}	101,40±24,14^{a}	3,19±0,78^{a}
	3 x 0.50	0,109±0,016^{a}	0,077±0,021^{a}	3,54±1,06^{a}	0,035±0,004^{a}	1,42±0,24^{a}	17,01±7,02^{a}	118,89±22,04^{ab}	3,13±0,74^{a}
					Effect of rootstock				
**P 22**		0,127±0,011^{b}	0,096±0,013^{b}	4,54±0,38^{b}	0,039±0,006^{b}	1,50±0,28^{a}	26,52±8,44^{b}	132,75±17,13^{b}	3,95±0,37^{b}
**P 60**		0,099±0,013^{a}	0,056±0,007^{a}	2,52±0,15^{a}	0,032±0,003^{a}	1,26±0,19^{a}	12,06±2,00^{a}	99,25±19,83^{a}	2,48±0,19^{a}
